# Impact of timing of adjuvant chemotherapy on survival in stage III colon cancer: a population-based study

**DOI:** 10.1186/s12885-018-4138-7

**Published:** 2018-03-01

**Authors:** Peng Gao, Xuan-zhang Huang, Yong-xi Song, Jing-xu Sun, Xiao-wan Chen, Yu Sun, Yu-meng Jiang, Zhen-ning Wang

**Affiliations:** 1 Department of Surgical Oncology and General Surgery, The First Hospital of China Medical University, 155 North Nanjing Street, Heping District Shenyang City, 110001 People’s Republic of China; 2 Department of Chemotherapy and Radiotherapy, The Second Affiliated Hospital and Yuying Children’s Hospital of Wenzhou Medical University, 109 Xueyuan West Road, Lucheng District, Wenzhou City, 325027 People’s Republic of China

**Keywords:** Colon cancer, Stage III, Timing of adjuvant chemotherapy, Postoperative complications, SEER-Medicare program

## Abstract

**Background:**

There is no consensus regarding the optimal time to initiate adjuvant chemotherapy after surgery for stage III colon cancer, and the relevant postoperative complications that cause delays in adjuvant chemotherapy are unknown.

**Methods:**

Eligible patients aged ≥66 years who were diagnosed with stage III colon cancer from 1992 to 2008 were identified using the linked Surveillance, Epidemiology, and End Results-Medicare database. Kaplan-Meier analysis and a Cox proportional hazards model were utilized to evaluate the impact of the timing of adjuvant chemotherapy on overall survival (OS).

**Results:**

A total of 18,491 patients were included. Delayed adjuvant chemotherapy was associated with worse OS (9–12 weeks: hazard ratio [HR] = 1.222, 95% confidence interval [CI] = 1.063–1.405; 13–16 weeks: HR = 1.252, 95% CI = 1.041–1.505; ≥ 17 weeks: HR = 1.969, 95% CI = 1.663–2.331). The efficacies of adjuvant chemotherapy within 5–8 weeks and ≤4 weeks were similar (HR = 1.045, 95% CI = 0.921–1.185). Compared with the non-chemotherapy group, chemotherapy initiated at ≥21 weeks did not significantly improve OS (HR = 0.882, 95% CI = 0.763–1.018). Patients with postoperative complications, particularly cardiac arrest, ostomy infection, shock, and septicemia, had a significantly higher risk of a 4- to 11-week delay in adjuvant chemotherapy (*p* < 0.05).

**Conclusions:**

Adjuvant chemotherapy initiated within 8 weeks was acceptable for patients with stage III colon cancer. Delayed adjuvant chemotherapy after 8 weeks was significantly associated with worse OS. However, adjuvant chemotherapy might still be useful even with a delay of approximately 5 months. Moreover, postoperative complications were significantly associated with delayed adjuvant chemotherapy.

## Background

Colon cancer is an important cause of cancer-related incidence and mortality and remains a major public health problem worldwide [1]. The current clinical practice guidelines from the National Comprehensive Cancer Network (NCCN) and the European Society for Medical Oncology (ESMO) recommend adjuvant chemotherapy following surgical resection as a standard treatment for patients with stage III colon cancer because of the benefit of chemotherapy in reducing the risk of recurrence and death by eradicating micrometastases [2].

Several studies have reported that the surgical resection of a primary tumor might induce angiogenesis and proliferation of dormant micrometastases by releasing growth-stimulating factors and triggering immunosuppression that leads to tumor growth [3–7]. Moreover, Harless et al. reported that the effectiveness of adjuvant chemotherapy was inversely proportional to the time from adjuvant chemotherapy initiation to surgical resection [8]. Therefore, it is a reasonable hypothesis that there may be a time-dependent cut-off point after surgery after which the benefit of adjuvant chemotherapy is not significant because of the failure to eradicate micrometastases. However, the NCCN and ESMO guidelines do not specify an optimal time to initiate adjuvant chemotherapy after surgical resection. Most clinical trials of adjuvant chemotherapy in colon cancer require adjuvant chemotherapy initiation within 6 to 8 weeks after surgical resection [9–12]. Routine preclinical and clinical data suggest that adjuvant chemotherapy in colon cancer should be initiated earlier rather than later, but, in real practice, the initiation of adjuvant chemotherapy in colon cancer is often delayed [13, 14].

There is no direct and high-quality evidence regarding the importance of the timing of adjuvant chemotherapy in colon cancer. Although two meta-analyses demonstrated that delays in the initiation of adjuvant chemotherapy were detrimental to survival in colorectal cancer [15, 16], these meta-analyses included both rectal and colon cancer, and it was thus not clear whether the conclusions could be applied to the treatment of colon cancer because of the biological differences between colon cancer and rectal cancer. To date, few retrospective studies evaluated the impact of the timing of adjuvant chemotherapy on survival in colon cancer, and the results were inconsistent [17–23]. Moreover, the relevant postoperative complications that cause delays in adjuvant chemotherapy are unknown.

Therefore, this population-based study was conducted to assess the impact of the timing of adjuvant chemotherapy on survival in stage III colon cancer and to assess whether postoperative complications were associated with the timing of adjuvant chemotherapy.

## Methods

### Data source

This study was conducted utilizing the Surveillance, Epidemiology, and End Results (SEER) program and Medicare-linked databases. The SEER program is a comprehensive source of population-based data on patient demographics, tumor characteristics, cancer-related treatments, and causes of death that covers approximately 28% of the population of the United States [24]. The Medicare database contains individual health insurance claims for approximately 97% of the population aged ≥65 years in the United States and complements the SEER with diagnoses, cancer-related treatments, and outcomes. In the Medicare database, Part A provides health-insurance data about hospitals, skilled-nursing facilities, hospices, and home health care, and Part B provides data about physician and outpatient services [25, 26]. The SEER-Medicare database was described in our previous study [27].

The access to the SEER-Medicare database was approved by National Cancer Institute and Information Management Services, Inc. (D6-MEDIC-821), and this study was approved by the Institutional Review Board of the First Hospital of China Medical University.

### Study population

This study included eligible patients aged ≥66 years from SEER-Medicare database who were diagnosed with primary colon adenocarcinoma from 1992 to 2008 (SEER cancer site codes 18.0, and 18.2 to 18.9). The participating patients fulfilled the American Joint Committee on Cancer (AJCC) staging criteria for stage III colon cancer and underwent primary tumor resection with curative intent within 180 days of diagnosis. The adjuvant chemotherapy regimens were 5-fluorourcil (5-FU)/capecitabine alone or 5-FU/capecitabine plus oxaliplatin (FOLFOX/CapeOX). The non-chemotherapy group included patients with no record of chemotherapy within one year of surgery. The FOLFOX/CapeOX group included patients with any record of 5-FU/capecitabine plus oxaliplatin within 4 weeks of their first chemotherapy dose.

The exclusion criteria were the following: (1) patients who previous non-colon cancer or a diagnosis of non-colon cancer within 1 year of the colon cancer diagnosis, (2) those with incomplete pathological stage entries or diagnostic data, (3) those who received adjuvant chemotherapy only after tumor relapse or metastasis, (4) those who received preoperative neoadjuvant treatments or other adjuvant chemotherapy regimens, (5) those who died within 30 days of diagnosis, and (6) those lacked full coverage from Medicare Parts A and B from 12 months before diagnosis to 9 months after diagnosis or were enrolled in a health maintenance organization.

The National Drug Codes for the drugs and the Health Care Financing Administration Common Procedure Coding System have been previously reported [27].

### Study variables

We obtained the patient demographics from the SEER patient entitlement and diagnosis summary file, including gender, age at diagnosis, race, marital status, residence location, household income, education level, and year of diagnosis. The disease characteristics, including primary tumor site (right-side or left-side colon), histologic grade (well differentiated, moderately differentiated, or poorly differentiated/undifferentiated), histologic type (adenocarcinoma, mucinous carcinoma, or signet-ring cell carcinoma), tumor stage, presence of preoperative obstruction or perforation, and number of examined lymph nodes (≥12 or < 12) were also studied. The tumor stage was assessed based on the seventh edition of the AJCC TNM staging system [28, 29]. The time to the initiation of adjuvant chemotherapy was defined as the interval between the curative surgery and the administration of the first chemotherapy.

For the evaluation of the comorbidities, we used the Hierarchical Condition Category (HCC) risk score to summarize the health care problems and predict the future health care cost of the population compared with the average Medicare beneficiary (HCC = 1.0), and the HCC risk score was derived from the Medicare inpatient and outpatient claims for various comorbidities within 12 months before the colon cancer diagnosis [30]. The postoperative complications were identified by assessing the discharge diagnoses within 1 month of surgery.

### Statistical analysis

For the descriptive analysis, the categorical variables were compared using χ2 tests and the continuous variables were compared using the Mann-Whitney U tests. In the univariate analysis of survival, Kaplan–Meier survival curves for overall survival (OS) were generated according to the chemotherapy regimen and timing of adjuvant chemotherapy, and these curves were compared using log-rank tests. A spline-based hazard ratio (HR) curve with the corresponding confidence limits was used to evaluate the effect of the continuous covariate of interest (i.e., the timing of adjuvant chemotherapy) on the outcome (OS) [31, 32]. Multivariate Cox proportional hazards models were used to determine the relationships of multiple survival-related variables with survival.

All statistical analyses were conducted using SAS version 9.3 (SAS Institute, Cary, NC, USA), STATA version 12.0 (Stata Corporation, College Station, TX, USA), SPSS version 18.0 (SPSS, Inc., Somers, NY, USA), and R version 3.1.1 (R Foundation for Statistical Computing, Vienna, Austria). For all analyses, a two-sided *p*-value of less than 0.05 was considered statistically significant.

## Results

### Patient characteristics

A total of 18,491 patients with stage III colon cancer who underwent surgical resection between 1992 and 2008 were identified using the SEER-Medicare database. Among these, 8058 patients received 5-FU or capecitabine alone, 1664 patients received FOLFOX, and 8769 patients did not receive adjuvant chemotherapy. With respect to the timing of adjuvant chemotherapy, 746 patients received adjuvant chemotherapy within 4 weeks after surgery, 6165 patients received adjuvant chemotherapy within 5–8 weeks after surgery, 1883 patients received adjuvant chemotherapy within 9–12 weeks after surgery, 466 patients received adjuvant chemotherapy within 13–16 weeks after surgery, and 462 patients received adjuvant chemotherapy ≥17 weeks after surgery. The patient profiles and disease characteristics are presented in Table 1.

**Table 1:** Clinicopathologic features of patients subjected to different chemotherapy regimens

	No-chemo	5FU/Capecitabine	FOLFOX/CapeOX
Gender			
Male	3124	3591	799
Female	5645	4467	865
Age at diagnosis, years			
66–70	554	1826	584
71–75	1002	2422	514
76–80	1784	2180	419
> 80	5429	1630	147
Race			
White	7369	6909	1407
Black	841	592	132
Asian	244	260	52
Other	315	297	73
Marital status			
Single+Separated	823	551	125
Married	3105	4620	1044
Divorced+Widowed	4535	2654	443
Other	306	233	52
Residence location			
Big Metro	4802	4219	878
Metro or Urban	2963	2853	588
Less Urban or Rural	1002	986	198
Median household income			
1st quartile	2203	1803	371
2nd quartile	2102	1976	375
3rd quartile	2062	1949	393
4th quartile	2035	2029	443
Unknown	367	301	82
Level of education			
1st quartile	2064	2003	401
2nd quartile	2029	2015	373
3rd quartile	2136	1920	400
4th quartile	2173	1819	408
Unknown	367	301	82
Year of diagnosis			
1992–1996	1837	1902	0
1997–2000	1678	1887	0
2001–2004	2739	3169	240
2005–2008	2515	1100	1424
Primary tumor site			
right-sided colon	5867	5111	1068
left-sided colon	2730	2809	572
unknown	172	138	24
Histologic grade			
Well	432	422	99
Moderate	5360	5169	1046
Poor+Undifferentiated	2756	2251	486
Unknown	221	216	33
Histologic type			
Adenocarcinoma	7402	6811	1425
Mucinous carcinoma	1216	1140	212
Signet-ring cell carcinoma	151	107	27
pT category			
pT1	173	302	69
pT2	571	721	150
pT3	6185	5805	1223
pT4a	1040	861	150
pT4b	800	369	72
pN category			
pN1a	3315	2898	500
pN1b	2889	2750	554
pN2a	1550	1518	357
pN2b	1015	892	253
pTNM stage			
pTNM IIIa	673	920	190
pTNM IIIb	6239	5814	1132
pTNM IIIc	1857	1324	342
Preoperative intestinal obstruction			
No	6406	6648	1367
Yes	2363	1410	297
Preoperative intestinal perforation			
No	8576	7998	1648
Yes	193	60	16
HCC risk score			
1st quartile	2557	1811	289
2nd quartile	1685	2427	539
3rd quartile	1972	2195	492
4th quartile	2555	1625	344
Number of examined lymph node			
≥ 12	4674	4274	1213
< 12	4095	3784	451
Postoperative radiotherapy			
No	8692	7765	1639
Yes	77	293	25
Timing to AC			
≤ 4 weeks	0	660	86
5–8 weeks	0	5118	1047
9–12 weeks	0	1502	381
13–16 weeks	0	369	97
≥ 17 weeks	0	409	53
No-chemo	8769	0	0

*Abbreviation: AC* Adjuvant chemotherapy, *HCC* Hierarchical Condition Categories; No-chemo, without adjuvant chemotherapy, *5-FU* 5-fluorouracil, *FOLFOX/CapeOX* 5-FU/capecitabine plus oxaliplatin

### Overall comparison of the timing of chemotherapy

**Fig. 1:**
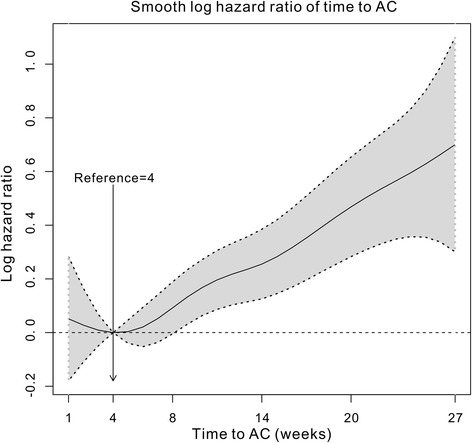
Splines-based hazard ratio curve for identification of the effect of timing of chemotherapy on overall survival. The solid line presents the relationship (log hazard ratio) between timing of chemotherapy and overall survival, and the dotted line presents the corresponding 95% confidence limits

**Fig. 2:**
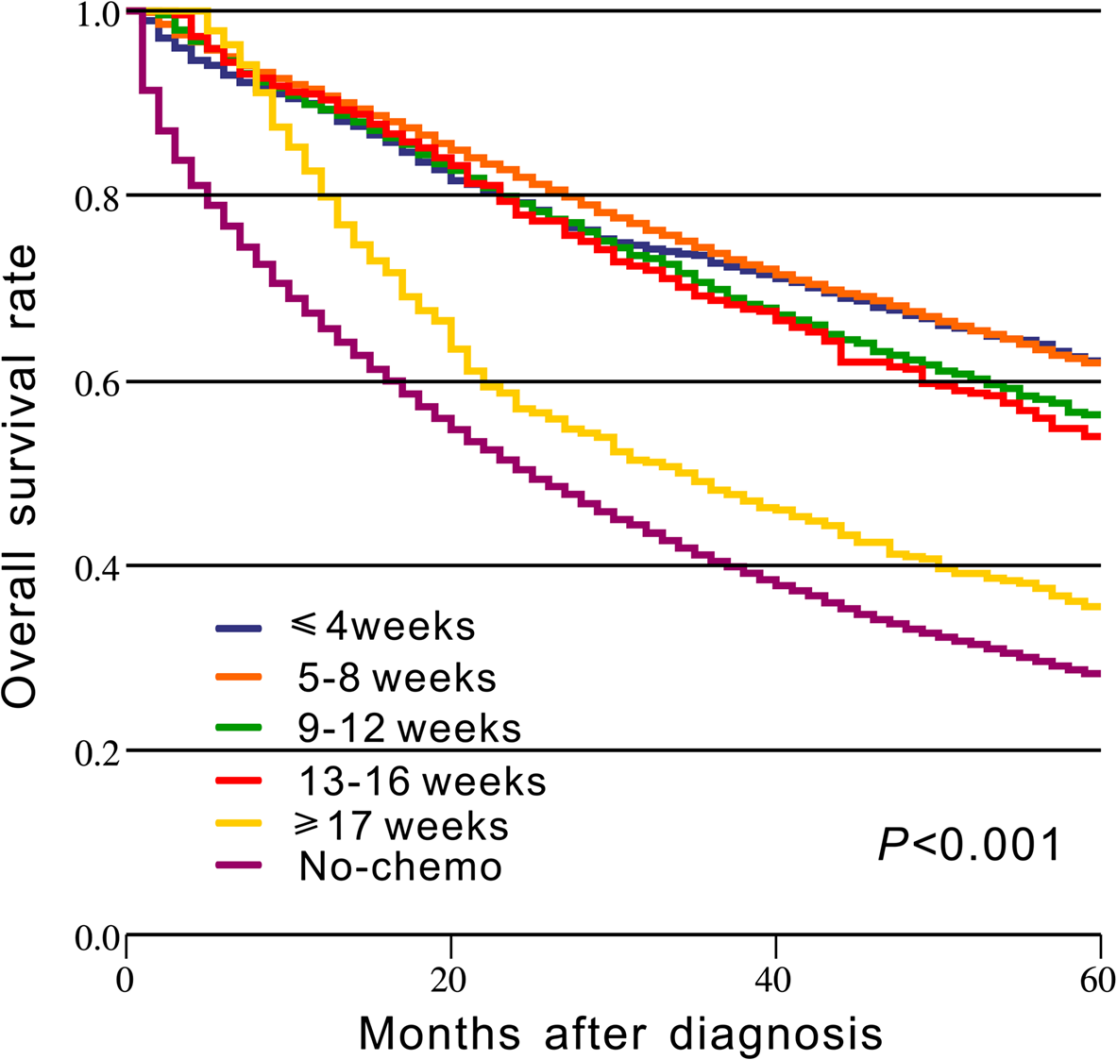
Kaplan–Meier curve of the timing of chemotherapy and overall survival. The *p* value is derived from log-rank test for the overall comparison of overall survival between different timing of chemotherapy and non-chemotherapy group

**Fig. 3:**
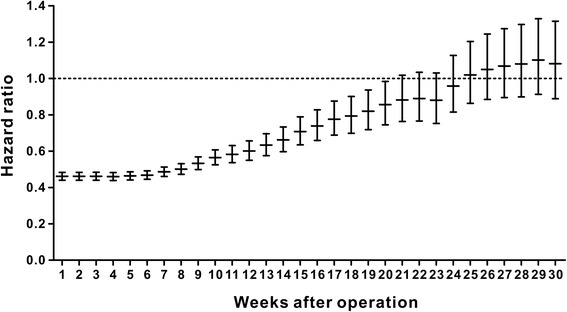
Hazard ratio plot for the relationship between timing of chemotherapy and overall survival compared with the non-chemotherapy group

We used a spline-based HR curve to explore the impact of the timing of adjuvant chemotherapy on overall survival in patients with stage III colon cancer. The results indicated that a minimum risk of mortality was achieved at 4 weeks after surgery, and the survival benefits decreased with a delay in the timing of adjuvant chemotherapy of more than 4 weeks (Fig. 1). Therefore, we used the value of ≤4 weeks as a reference for the survival analysis, and the results of univariate analyses indicated that delayed chemotherapy was significantly associated with worse OS (9–12 weeks: HR = 1.169, 95% confidence interval [CI] = 1.019–1.341, *p* = 0.026; 13–16 weeks: HR = 1.237, 95% CI = 1.031–1.483, *p* = 0.022; ≥ 17 weeks: HR = 2.207, 95% CI = 1.870–2.604, *p* < 0.001). However, chemotherapy that was initiated within 5–8 weeks after surgery did not significantly increase the risk of mortality (HR = 0.982, 95% CI = 0.867–1.113, *p* = 0.780). A Kaplan–Meier survival curve that was stratified by the timing of chemotherapy is presented in Fig. 2. Multivariate Cox proportional hazards models produced results similar to those of the univariate analyses (5–8 weeks: HR = 1.045, 95% CI = 0.921–1.185, *p* = 0.498; 9–12 weeks: HR = 1.222, 95% CI = 1.063–1.405, *p* = 0.005; 13–16 weeks: HR = 1.252, 95% CI = 1.041–1.505, *p* = 0.017; ≥ 17 weeks: HR = 1.969, 95% CI = 1.663–2.331, p < 0.001, Table 2). Moreover, the survival benefit of adjuvant chemotherapy was statistically insignificant when adjuvant chemotherapy was initiated ≥21 weeks after resection compared with the non-chemotherapy group (HR = 0.882, 95% CI = 0.763–1.018, *p* = 0.087, Fig. 3), and chemotherapy initiated ≥25 weeks after surgery did not elicit an OS benefit compared with the non-chemotherapy group (HR = 1.019, 95% CI = 0.863–1.204, *p* = 0.821, Fig. 3).

**Table 2:** Univariate and multivariate Cox proportional hazards analysis of factors influencing the 5-year overall survival for patients who underwent chemotherapy

Variables	Univariate analysis			Multivariate analysis^*^		
	HR	95% CI	*P*	HR	95% CI	*P*
Gender			0.136			
Male	1					
Female	0.952	0.893–1.015				
Age at diagnosis, years			< 0.001			< 0.001
66–70	1			1		
71–75	1.157	1.054–1.269		1.133	1.030–1.245	
76–80	1.359	1.238–1.492		1.330	1.209–1.463	
> 80	1.929	1.752–2.123		1.834	1.657–2.029	
Race			< 0.001			0.001
White	1			1		
Black	1.039	0.922–1.171		0.980	0.864–1.112	
Asian	0.625	0.503–0.777		0.636	0.511–0.793	
Other	0.989	0.836–1.169		0.961	0.811–1.139	
Marital status			< 0.001			0.011
Single+Separated	1			1		
Married	0.818	0.723–.926		0.856	0.755–0.970	
Divorced+Widowed	0.994	0.874–1.129		0.948	0.833–1.079	
Other	0.838	0.670–1.047		0.905	0.723–1.133	
Residence location			0.222			
Big Metro	1					
Metro or Urban	0.942	0.878–1.010				
Less Urban or Rural	0.996	0.901–1.102				
Median household income			0.023			0.872
1st quartile	1			1		
2nd quartile	0.993	0.906–1.088		1.042	0.943–1.152	
3rd quartile	0.926	0.843–1.016		1.000	0.895–1.119	
4th quartile	0.872	0.795–0.957		1.013	0.893–1.15	
Unknown	0.945	0.795–1.123		1.056	0.860–1.298	
Level of education			< 0.001			0.001
1st quartile	1			1		
2nd quartile	1.164	1.061–1.278		1.154	1.045–1.274	
3rd quartile	1.159	1.055–1.272		1.142	1.022–1.276	
4th quartile	1.262	1.149–1.385		1.286	1.130–1.463	
Unknown	1.142	0.960–1.358		N/A^a^	N/A^a^	
Year of diagnosis			< 0.001			< 0.001
1992–1996	1			1		
1997–2000	0.863	0.784–0.950		0.833	0.756–0.918	
2001–2004	0.814	0.748–0.885		0.754	0.692–0.822	
2005–2008	0.667	0.605–0.737		0.609	0.549–0.675	
Primary tumor site			< 0.001			0.006
right-sided colon	1			1		
left-sided colon	0.822	0.767–0.880		0.891	0.829–0.957	
unknown	1.241	0.988–1.559		1.034	0.821–1.302	
Histologic grade			< 0.001			< 0.001
Well	1			1		
Moderate	1.156	0.987–1.353		1.073	0.915–1.257	
Poor+Undifferentiated	1.748	1.487–2.055		1.384	1.174–1.630	
Unknown	1.274	0.991–1.638		1.120	0.869–1.445	
Histologic type			< 0.001			0.101
Adenocarcinoma	1			1		
Mucinous carcinoma	1.123	1.026–1.229		1.024	0.934–1.123	
Signet-ring cell carcinoma	1.893	1.503–2.384		1.289	1.019–1.632	
pT category			< 0.001			< 0.001
pT1	1			1		
pT2	1.071	0.824–1.391		1.019	0.783–1.325	
pT3	2.025	1.616–2.536		1.594	1.269–2.002	
pT4a	2.98	2.348–3.782		2.205	1.732–2.806	
pT4b	5.459	4.253–7.008		3.404	2.636–4.395	
pN category			< 0.001			< 0.001
pN1a	1			1		
pN1b	1.374	1.263–1.495		1.305	1.199–1.420	
pN2a	1.844	1.682–2.021		1.675	1.526–1.838	
pN2b	3.215	2.920–3.541		2.874	2.595–3.183	
Preoperative intestinal obstruction			< 0.001			< 0.001
No	1			1		
Yes	1.425	1.319–1.540		1.246	1.152–1.349	
Preoperative intestinal perforation			< 0.001			0.001
No	1			1		
Yes	2.284	1.723–3.028		1.628	1.223–2.168	
HCC risk score			< 0.001			< 0.001
1st quartile	1			1		
2nd quartile	0.950	0.865–1.043		1.161	1.053–1.280	
3rd quartile	1.109	1.010–1.217		1.347	1.223–1.483	
4th quartile	1.447	1.315–1.593		1.644	1.489–1.815	
Number of examined lymph node			0.003			< 0.001
≥ 12	1			1		
< 12	1.102	1.034–1.175		1.295	1.209–1.387	
Postoperative radiotherapy			< 0.001			< 0.001
No	1			1		
Yes	1.620	1.391–1.887		1.323	1.133–1.545	
Timing to AC			< 0.001			< 0.001
≤ 4 weeks	1			1		
5–8 weeks	0.982	0.867–1.113		1.045	0.921–1.185	
9–12 weeks	1.169	1.019–1.341		1.222	1.063–1.405	
13–16 weeks	1.237	1.031–1.483		1.252	1.041–1.505	
≥ 17 weeks	2.207	1.870–2.604		1.969	1.663–2.331	

*Abbreviation*: *HR* Hazard ratio, *CI* Confidence interval, *HCC* Hierarchical Condition Categories, *AC* Adjuvant chemotherapy

^*^Only variables with a *p* < 0.05 in the univariate analysis were included in the multivariate analysis

^a^unavailable because of colinearity with the variable of Median household income

### Comparison of the timing of FOLFOX/CapeOX chemotherapy

**Fig. 4:**
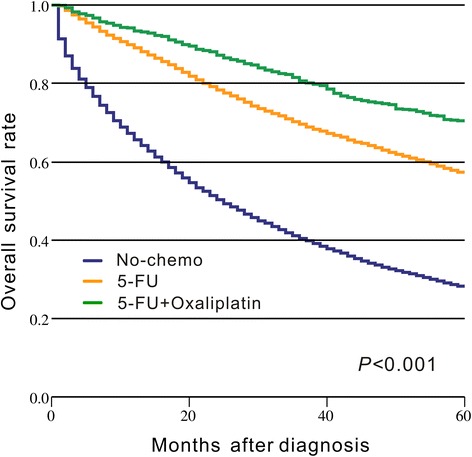
Kaplan–Meier curve of chemotherapy regimen and overall survival. The p value is derived from log-rank test for the overall comparison of overall survival between different chemotherapy regimens and non-chemotherapy group

**Fig. 5:**
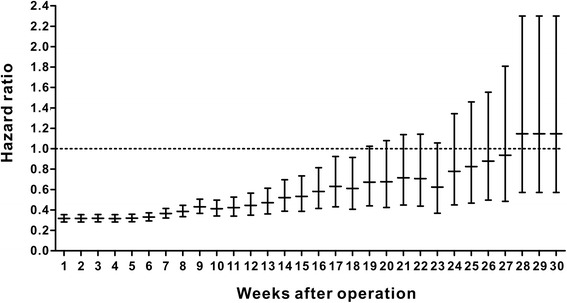
Hazard ratio plot for the relationship between timing of FOLFOX/CapeOX chemotherapy and overall survival compared with the non-chemotherapy group

Our results indicated that the survival benefit from FOLFOX/CapeOX chemotherapy was more evident than that from 5-FU alone in patients with stage III colon cancer (HR = 0.615, 95% CI = 0.555–0.683, *p* < 0.001, Fig. 4), although both chemotherapy regimens significantly improved the OS (p < 0.001) compared with the non-chemotherapy group. Therefore, the relationship between the timing of FOLFOX/CapeOX chemotherapy and OS was further evaluated. The results of the multivariate analysis indicated that FOLFOX/CapeOX chemotherapy that was initiated within 5–8 weeks did not increase the risk of mortality compared with FOLFOX/CapeOX chemotherapy that was initiated ≤4 weeks after surgery (HR = 1.009, 95% CI = 0.619–1.644, *p* = 0.971, Table 3). However, FOLFOX/CapeOX chemotherapy initiated within 9–12, 13–16, and ≥17 weeks tended to produce worse OS (9–12 weeks: HR = 1.640, 95% CI = 0.990–2.717, *p* = 0.055; 13–16 weeks: HR = 1.422, 95% CI = 0.788–2.566, *p* = 0.243; ≥ 17 weeks: HR = 2.482, 95% CI = 1.354–4.549, *p* = 0.003, Table 3). Indeed, the spline-based HR curve for FOLFOX/CapeOX chemotherapy indicated that the survival benefit of FOLFOX/CapeOX chemotherapy was not statistically significant when it was initiated at ≥19 weeks compared with the non-chemotherapy group (HR = 0.672, 95% CI = 0.441–1.024, *p* = 0.064, Fig. 5).

**Table 3:** Univariate and multivariate Cox proportional hazards analysis of factors influencing 5-year overall survival for patients who underwent FOLFOX/CapeOX chemotherapy

Variables	Univariate analysis			Multivariate analysis^*^		
	HR	95% CI	*P*	HR	95% CI	*P*
Gender			0.092			
Male	1					
Female	0.845	0.695–1.028				
Age at diagnosis, years			0.003			0.007
66–70	1			1		
71–75	1.122	0.872–1.443		1.149	0.888–1.486	
76–80	1.286	0.994–1.665		1.293	0.991–1.688	
> 80	1.812	1.305–2.517		1.816	1.285–2.566	
Race			0.206			
White	1					
Black	1.028	0.719–1.469				
Asian	1.156	0.677–1.972				
Other	0.502	0.259–0.973				
Marital status			0.167			
Single+Separated	1					
Married	1.036	0.696–1.543				
Divorced+Widowed	1.318	0.869–1.999				
Other	1.066	0.540–2.104				
Residence location			0.329			
Big Metro	1					
Metro or Urban	1.071	0.865–1.327				
Less Urban or Rural	1.252	0.929–1.686				
Median household income			0.007			0.497
1st quartile	1			1		
2nd quartile	0.962	0.733–1.261		1.021	0.755–1.380	
3rd quartile	0.745	0.561–0.989		0.814	0.575–1.152	
4th quartile	0.620	0.465–0.826		0.792	0.529–1.186	
Unknown	0.800	0.496–1.291		0.947	0.514–1.743	
Level of education			0.006			0.263
1st quartile	1			1		
2nd quartile	1.524	1.117–2.079		1.371	0.979–1.920	
3rd quartile	1.552	1.147–2.100		1.379	0.960–1.982	
4th quartile	1.744	1.298–2.343		1.289	0.854–1.947	
Unknown	1.417	0.863–2.327		N/A^a^	N/A^a^	
Year of diagnosis			0.398			
2001–2004	1					
2005–2008	0. 897	0.697–1.154				
Primary tumor site			0.150			
right-sided colon	1					
left-sided colon	0.878	0.711–1.084				
unknown	1.602	0.824–3.114				
Histologic grade			< 0.001			0.022
Well	1			1		
Moderate	1.170	0.724–1.892		1.015	0.623–1.653	
Poor+Undifferentiated	1.973	1.211–3.215		1.407	0.856–2.315	
Unknown	1.376	0.598–3.165		0.987	0.422–2.309	
Histologic type			0.008			0.148
Adenocarcinoma	1			1		
Mucinous carcinoma	1.491	1.146–1.940		1.306	0.997–1.712	
Signet-ring cell carcinoma	1.467	0.728–2.959		1.147	0.556–2.366	
pT category			< 0.001			< 0.001
pT1	1			1		
pT2	1.374	0.499–3.780		1.472	0.531–4.080	
pT3	3.645	1.506–8.823		2.730	1.118–6.667	
pT4a	6.221	2.494–15.521		5.077	2.014–12.801	
pT4b	7.165	2.766–18.559		4.350	1.656–11.424	
pN category			< 0.001			< 0.001
pN1a	1			1		
pN1b	1.581	1.172–2.132		1.475	1.090–1.996	
pN2a	2.301	1.691–3.132		1.970	1.440–2.696	
pN2b	4.310	3.195–5.814		3.408	2.497–4.650	
Preoperative intestinal obstruction			< 0.001			0.055
No	1			1		
Yes	1.680	1.340–2.106		1.258	0.995–1.590	
Preoperative intestinal perforation			0. 165			
No	1					
Yes	1.770	0.790–3.966				
HCC risk score			< 0.001			< 0.001
1st quartile	1			1		
2nd quartile	0.936	0.683–1.283		1.129	0.816–1.561	
3rd quartile	1.033	0.754–1.415		1.273	0.918–1.765	
4th quartile	1.994	1.469–2.705		2.197	1.592–3.033	
Number of examined lymph node			0.382			
≥ 12	1					
< 12	0.906	0.727–1.130				
Postoperative radiotherapy			0.055			
No	1					
Yes	1.850	0.988–3.467				
Timing to AC			< 0.001			< 0.001
≤ 4 weeks	1			1		
5–8 weeks	1.028	0.635–1.663		1.009	0.619–1.644	0.971
9–12 weeks	1.665	1.012–2.739		1.640	0.990–2.717	0.055
13–16 weeks	1.671	0.935–2.988		1.422	0.788–2.566	0.243
≥ 17 weeks	3.144	1.731–5.710		2.482	1.354–4.549	0.003

*Abbreviation FOLFOX/CapeOX* 5-FU/capecitabine plus oxaliplatin, *HR* Hazard ratio, *CI* Confidence interval, *HCC* Hierarchical Condition Categories, *AC* Adjuvant chemotherapy

^*^Only variables with a *p* < 0.05 in the univariate analysis were included in the multivariate analysis

^a^unavailable because of colinearity with the variable of Median household income

### Postoperative complications and the timing of chemotherapy

**Fig. 6:**
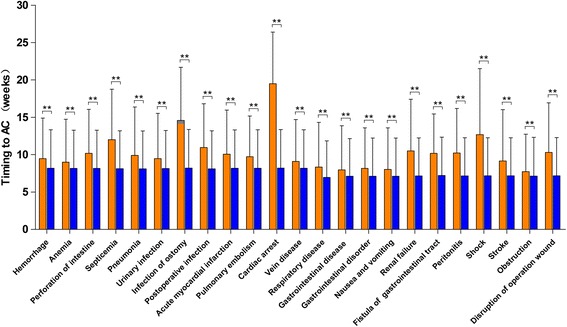
Association between postoperative complications and timing of adjuvant chemotherapy (AC) after surgical resection. Orange color bars present the timing of AC among patients with postoperative complications. Blue color bars present the timing of AC among patients without postoperative complications. “**” present a significant difference with *p* value < 0.01

We examined the correlation of postoperative complications with the delayed initiation of adjuvant chemotherapy. The results indicated that patients with postoperative complications had a significantly higher risk of delayed adjuvant chemotherapy (*p* < 0.05; Fig. 6). Among the postoperative complications, cardiac arrest (19.50 vs. 8.22 weeks; Δ = 11.28 weeks), ostomy infection (14.60 vs. 8.22 weeks; Δ = 6.38 weeks), shock (13.69 vs. 8.18 weeks; Δ = 5.51 weeks), and septicemia (12.02 vs. 8.13 weeks; Δ = 3.89 weeks) had strong influences on chemotherapy delay with a delay of approximately 4–11 weeks. Additionally, disruption of the operation wound (Δ = 3.11 weeks), peritonitis (Δ = 3.07 weeks), fistula of the gastrointestinal tract (Δ = 2.97 weeks), acute renal failure (Δ = 3.34 weeks), postoperative infection (Δ = 2.85 weeks), intestinal perforation (Δ = 2.02 weeks), acute myocardial infarction (Δ = 1.88 weeks), and stroke (Δ = 1.96 weeks) could result in delays in the initiation of adjuvant chemotherapy of approximately 2–3 weeks. In turn, hemorrhage, pneumonia, urinary infection, pulmonary embolism, respiratory disease, gastrointestinal disorder, anemia, vein disease, gastrointestinal disease, nausea and vomiting, and obstruction had relatively weak impacts on the chemotherapy delay (a delay of approximately 0.5–1.5 weeks), although the differences were significant.

## Discussion

There is no evidence about the optimal time to initiate adjuvant chemotherapy after surgical resection, or whether there is an ideal timing for adjuvant therapy after which treatment benefit decreases. This population-based study based on the SEER-Medicare databases was conducted to evaluate the relationship between the timing of adjuvant chemotherapy and survival in stage III colon cancer. The results indicated that adjuvant chemotherapy that was initiated within 5–8 weeks after surgery did not increase the risk of mortality compared with chemotherapy initiated at ≤4 weeks after surgery, and the initiation of adjuvant chemotherapy within 8 weeks after surgery was thus feasible. However, adjuvant chemotherapy after 8 weeks of surgery was significantly associated with worse OS. The survival benefit of adjuvant chemotherapy became statistically insignificant when chemotherapy was initiated after 21 weeks compared with the non-chemotherapy group, thus, adjuvant chemotherapy might be still useful even with a delay of approximately 5 months (Fig. 3). Our results indicated that the survival benefits of the FOLFOX/CapeOX chemotherapy regimen within 5–8 weeks and ≤4 weeks were similar, and chemotherapy initiated ≥19 weeks did not have a significant OS benefit compared with the non-chemotherapy group.

The favorable effect of adjuvant chemotherapy on survival primarily involves the eradication of residual disease and micrometastases. However, the relationship between the timing of adjuvant chemotherapy and survival is unclear. Several studies reported that primary tumor removal could accelerate angiogenesis and growth of residual disease and micrometastases by releasing growth-stimulating factors and promoting immunosuppression [3–7]; thus, a delay in adjuvant chemotherapy might favor tumor angiogenesis and growth, and a long delay could lead to tumor recurrence or metastasis and a consequent failure to achieve the curative potential of adjuvant chemotherapy. Furthermore, Goldie et al. suggested that the drug sensitivity of a tumor was related to the spontaneous mutation rate toward phenotypic drug resistance, which was a function of time [33]. Moreover, the mathematical model by Harless et al. demonstrated that the effectiveness of chemotherapy was inversely proportional to the tumor burden that had to be eradicated, which, in turn, was a function of the time of the initiation of chemotherapy after surgery [8]. Therefore, the survival benefit of adjuvant chemotherapy was time-dependent. Studies have also reported that delayed chemotherapy might reflect poor patient and disease characteristics and increase comorbidity, which would be associated with poor prognoses [13, 34].

Our spline-based HR model revealed that the efficacies of adjuvant chemotherapy within 5–8 weeks and ≤4 weeks were similar, although the minimum risk of mortality was achieved at 4 weeks after surgery. Bos et al. demonstrated that adjuvant chemotherapy within 5–6 weeks or 7–8 weeks after surgery did not decrease OS compared to the initiation of chemotherapy within 4 weeks, and the start of chemotherapy 8 weeks after surgery was associated with a decreased OS [35]. In clinical practice, it is important to note that the toxicity of chemotherapy may be maximized due to poor immune and performance statuses after surgery, and thus, initiating chemotherapy early may cause severe chemotherapy-related adverse events and even death [36]. Therefore, an additional survival benefit of excess-early adjuvant chemotherapy may be difficult to detect because of the severe adverse events caused by chemotherapy. The initiation of adjuvant chemotherapy within 8 weeks after surgery was feasible. However, adjuvant chemotherapy that was initiated ≥21 weeks after surgery did not have a significant survival OS benefit compared with the non-chemotherapy group, and conversely, this delay may cause additional chemotherapy-related adverse events. Further studies are needed to explore the optimal timing for adjuvant chemotherapy, for example, identifying the time at which the survival benefit from chemotherapy maximally outweighs the risks of chemotherapy-related adverse events and death.

Several studies reported that patient and disease characteristics, including older age, low income, and high comorbidity, were associated with delayed adjuvant chemotherapy [13, 34]. Cheung et al. reported that the determinants of delayed adjuvant chemotherapy might be primarily influenced by their relationships with the postoperative complications that ultimately resulted in chemotherapy delay, and these complications seemed to be a more important driver for chemotherapy delay [37]. Therefore, the relationship between postoperative complications and delayed adjuvant chemotherapy was evaluated, and the results indicated that patients with postoperative complications had a significantly higher risk of delayed adjuvant chemotherapy (*p* < 0.05). Specifically, cardiac arrest, ostomy infection, shock, and septicemia had strong influences on delayed chemotherapy and caused delays of 4–11 weeks. Moreover, disruption of the operation wound, peritonitis, fistula of the gastrointestinal tract, acute renal failure, postoperative infection, intestinal perforation, acute myocardial infarction, and stroke could cause delays of 2–3 weeks. These results were expected because patients with severe postoperative complications were likely to require more time for recovery. Therefore, multidisciplinary treatment strategies are needed to reduce postoperative complications and promote timely adjuvant chemotherapy.

This study has limitations. First, this was a retrospective SEER-Medicare study, and thus the potential for confounding based on patient selection could not be completely eliminated. Second, the data on the patient/disease characteristics and treatments were obtained from a fee-for-service insurance database. Some clinical variables were not available, and the presence of other important confounding factors could not be discarded. Third, the use of adjuvant chemotherapy may decrease in older patients mainly because older patients are more likely to have high comorbidity and poor performance statuses, and oncologists may be less willing to use adjuvant chemotherapy [38, 39]. In our study, the results demonstrated that the use of adjuvant chemotherapy was common in older patients with stage III colon cancer (9722/18,491, 52.6%), and adjuvant chemotherapy significantly improved the prognoses compared with the non-chemotherapy group. Additionally, several studies have also demonstrated that older patients with stage III colon cancer gain a significant survival benefit from adjuvant chemotherapy [40–43]. Therefore, further large-scale, high-quality studies are needed to evaluate the interactions of age and the timing of adjuvant chemotherapy with survival in stage III colon cancer. Fourth, disease-free survival was also an appropriate measure for assessing the survival benefit of adjuvant chemotherapy; however, disease-free survival could not be evaluated because the data on disease-free survival was not available in the SEER-Medicare database. Further studies are required to investigate the impact of the timing of adjuvant chemotherapy on disease-free survival. Moreover, it was not feasible to conduct a randomized controlled trial to specifically address the impact of the timing of adjuvant chemotherapy on survival in colon cancer. Thus, larger-scale and well-designed retrospective studies are needed to explore the optimal timing of adjuvant chemotherapy after surgical resection.

## Conclusions

The survival benefits of adjuvant chemotherapy within 5–8 weeks and ≤4 weeks were similar, and thus, initiation of adjuvant chemotherapy within 8 weeks in patients with stage III colon cancer was feasible. Adjuvant chemotherapy 8 weeks after surgical resection was significantly associated with worse OS. However, adjuvant chemotherapy might still be useful even with a delay of approximately 5 months, although the survival benefit was reduced. Additionally, postoperative complications were significantly associated with the delayed initiation of adjuvant chemotherapy in patients with stage III colon cancer.

## Abbreviations

5-FU: 5-fluorourcil
AJCC: American Joint Committee on Cancer
CIs: Confidence intervals
ESMO: The European Society for Medical Oncology
HCC: Hierarchical Condition Category.
HR: Hazard ratio.
NCCN: The National Comprehensive Cancer Network.
OS: Overall survival.
SEER: Surveillance, Epidemiology, and End Results
